# Autoimmune uveitis in Behçet's disease and Vogt‐Koyanagi‐Harada disease differ in tissue immune infiltration and T cell clonality

**DOI:** 10.1002/cti2.1461

**Published:** 2023-09-15

**Authors:** Hao Kang, Hongjian Sun, Yang Yang, Zewen K Tuong, Minglei Shu, Yunbo Wei, Yu Zhang, Di Yu, Yong Tao

**Affiliations:** ^1^ Department of Ophthalmology, Beijing Chaoyang Hospital Capital Medical University Beijing China; ^2^ Frazer Institute, Faculty of Medicine The University of Queensland Brisbane QLD Australia; ^3^ Shandong Artificial Intelligence Institute Qilu University of Technology (Shandong Academy of Sciences) Jinan China; ^4^ Ian Frazer Centre for Children's Immunotherapy Research, Children's Health Research Centre, Faculty of Medicine The University of Queensland Brisbane QLD Australia; ^5^ School of Pharmaceutical Sciences, Laboratory of Immunology for Environment and Health, Shandong Analysis and Test Center Qilu University of Technology (Shandong Academy of Sciences) Jinan China

**Keywords:** Behcet disease, single‐cell RNA sequencing, T‐cell clonality, uveitis, Vogt‐Koyanagi‐Harada disease

## Abstract

**Objectives:**

Non‐infectious uveitis is often secondary to systemic autoimmune diseases, with Behçet's disease (BD) and Vogt‐Koyanagi‐Harada disease (VKHD) as the two most common causes. Uveitis in BD and VKHD can show similar clinical manifestations, but the underlying immunopathogenesis remains unclear.

**Methods:**

To understand immune landscapes in inflammatory eye tissues, we performed single‐cell RNA paired with T cell receptor (TCR) sequencing of immune cell infiltrates in aqueous humour from six patients with BD (*N* = 3) and VKHD (*N* = 3) uveitis patients.

**Results:**

Although T cells strongly infiltrated in both types of autoimmune uveitis, myeloid cells only significantly presented in BD uveitis but not in VKHD uveitis. Conversely, VKHD uveitis but not BD uveitis showed an overwhelming dominance by CD4^+^ T cells (> 80%) within the T cell population due to expansion of CD4^+^ T cell clusters with effector memory (Tem) phenotypes. Correspondingly, VKHD uveitis demonstrated a selective expansion of CD4^+^ T cell clones which were enriched in pro‐inflammatory Granzyme H^+^ CD4^+^ Tem cluster and showed TCR and Th1 pathway activation. In contrast, BD uveitis showed a preferential expansion of CD8^+^ T cell clones in pro‐inflammatory Granzyme H^+^ CD8^+^ Tem cluster, and pathway activation for cytoskeleton remodelling, cellular adhesion and cytotoxicity.

**Conclusion:**

Single‐cell analyses of ocular tissues reveal distinct landscapes of immune cell infiltration and T‐cell clonal expansions between VKHD and BD uveitis. Preferential involvements of pro‐inflammatory CD4^+^ Th1 cells in VKHD and cytotoxic CD8^+^ T cells in BD suggest a difference in disease immunopathogenesis and can guide precision disease management.

## Graphical abstract

Single‐cell analyses of ocular tissues reveal distinct landscapes of immune cell infiltration and T‐cell clonal expansions between Behçet's disease (BD) and Vogt‐Koyanagi‐Harada disease (VKHD) uveitis. Preferential involvements of pro‐inflammatory CD4^+^ Th1 cells in VKHD and cytotoxic CD8^+^ T cells in BD suggest a difference in disease immunopathogenesis and can guide precision disease management.

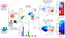

## Introduction

Uveitis is an inflammatory disorder of the uveal tract of the eye that can be caused by multiple reasons. This vision‐threatening disease can lead to serious complications such as visual impairment and blindness in about 35% of patients.^1^ Uveitis is divided into infectious and non‐infectious types according to its aetiology. As a more common type, non‐infectious uveitis is often related to autoimmune diseases, such as Behçet's disease (BD) and Vogt‐Koyanagi‐Harada disease (VKHD). Although BD and VKHD show the characteristics of familial aggregation, they differ in geographical distribution. BD is more prevalent in Asia, the Middle East and the Mediterranean than in other regions and is characterised by three main clinical features of non‐granulomatous uveitis, oral ulcers and genital ulcers.^2^ VKHD is considered to distribute worldwide, with a higher occurrence in people with darker skin.^3^ The clinical features of VKHD are bilateral granulomatous pancreatitis, polio, vitiligo and central nervous system abnormalities.^4^

The immunological pathogenesis of BD and VKHD remains elusive although the dysregulated function of T cells, including cytotoxic CD8^+^ T cells or CD4^+^ T cell subset Th1 and Th17 cells, have been implicated in the development of both BD and VKHD.^5^, ^6^ Transcriptomic profiling of iris specimens from patients with BD uveitis revealed that T cell activation was the most significantly enriched biological process.^7^ Active BD uveitis showed significant intraocular infiltration of CD8^+^ T cells and an increased proportion of natural killer T (NKT) cells.^8^ The innate immune system was also reported in a hyperactive state in BD patients, showing enhanced pro‐inflammation by neutrophils, monocytes or natural killer (NK) cells.^9^ Analysing peripheral blood mononuclear cells (PBMCs) also revealed that monocytes, especially the C1Q^hi^ monocyte subtype, exhibited significant expansion and pro‐inflammatory characteristics, which was associated with clinical disease activities in BD.^10^

VKHD is widely acknowledged as a T cell‐mediated autoimmune disorder that directs against melanocyte or melanocyte‐associated antigens.^11^ VKHD patients had significantly higher numbers of T cells with overactivated and more differentiated phenotypes in PBMCs.^12^ Analyses of peripheral blood by flow cytometry and cytokine profiles by enzyme‐linked immunosorbent assay suggested polarised Th1 and/or Th17 cells in VKHD.^13^ Dysregulation of circulating monocytes was also found in VKHD, suggesting enhanced antigen presentation and pro‐inflammatory functions in VKHD.^14^

Although these studies provide important information for the immune dysregulation in autoimmune uveitis in BD and VKHD, there is a lack of information on immune responses in inflammatory ocular tissues. To gain insights into the immunological pathogenesis of autoimmune uveitis and specifically ask the question of specific immune signatures of autoimmune uveitis selectively associated with BD or VKHD, we performed single‐cell RNA sequencing (scRNA‐seq) of cellular infiltrates in aqueous humour from a cohort of six uveitis patients associated with BD (*N* = 3) or VKHD (*N* = 3). We found significant T‐cell infiltration in both BD and VKHD uveitis, but the infiltration of myeloid cells was largely confined to BD uveitis. Single‐cell T‐cell receptor sequencing (scTCR‐seq) further demonstrated a strong clonal expansion of effector memory CD4^+^ T cells in VKHD uveitis that expressed pro‐inflammatory cytokines featured by Th1 cytokine *IFNG*. In contrast, BD uveitis showed a selective clonal expansion of effector memory CD8^+^ T cells expressing cytotoxic molecules such as *GZMB*. Therefore, tissue immune infiltrates and T‐cell clonalities are distinctively associated with BD and VKHD, thus suggesting different approaches in managing two types of autoimmune uveitis.

## Results

### T cells are the dominant immune cell type infiltrating in uveitis lesions but myeloid cells also present in BD uveitis

Six uveitis patients (3 BD, 1F/2M, 14–38 years old; 3 VKHD, 2F/1M, 8–39 years old) volunteered to participate in this study. They all had decreased vision and four of them (2 BD and 2 VKHD) had cataract surgery before sample collections. All patients underwent immunosuppressive treatments but there were no major differences between the two groups. Full demographic and clinical information are shown in Table 1.

**Table 1:** Patient information summary

Patient	VKHD1	VKHD2	VKHD3	BD1	BD2	BD3
Age at collection	34	39	8	38	26	14
Gender	Female	Male	Female	Female	Male	Male
Onset year	2016	2005	2022	2015	2020	2021
Year of sampling	2019	2019	2023	2019	2023	2023
Ocular symptom	Decreased vision in bilateral eyes with accompanying blurred vision	Decreased vision in bilateral eyes	Decreased vision in bilateral eyes with accompanying red‐eye	Decreased vision in left eye with accompanying red‐eye	Decreased vision in left eye with accompanying red‐eye and eye pain	Decreased vision in bilateral eyes
Visual acuity	20/125 OD^a^ and 20/50 OS^b^	20/320 OD and 20/30 OS	20/63 OD and 20/40 OS	No light perception OD and 20/32 OS	20/20 OD and 20/32 OS	20/63 OD and 20/50 OS
Anterior segment slit‐lamp examination	Bilateral recurrent anterior uveitis (mutton fat KP^c^, aqueous flare 2+ and anterior chamber cells 3+ OU^d^)	Bilateral recurrent anterior uveitis (mutton fat KP, aqueous flare + and anterior chamber cells 2+ OU)	Bilateral anterior uveitis (mutton fat KP, aqueous flare + and anterior chamber cells +, posterior synechiae OU)	Bilateral recurrent anterior uveitis (fine, non‐pigmented KP OD and no KP was observed in OS, aqueous flare 2+ and anterior chamber cells 3+ OU)	Recurrent anterior uveitis of the left eye (fine, no‐pigmented KP, aqueous flare 3+ and anterior chamber cells 3+ OS)	Bilateral recurrent anterior uveitis (fine, non‐pigmented KP, aqueous flare + and anterior chamber cells + OU)
Fundus examination	Sunset glow fundus, Dalen‐Fuchs nodules and retinal pigment epithelium clumping and migration	Sunset glow fundus	Vitreous cells 2+, hyperemic disc, multiple circumscribed retinal oedema in OU	Optic atrophy, vascular occlusion and gliotic sheathing in OD; retinal vasculitis and retinal oedema in OS	Severe vitritis in OS, retinal vasculitis and retinal oedema in OS	Severe vitritis in OD and moderate vitirtis in OS, retinal vasculitis and mild optic disc oedema in OU
Surgery	Cataract surgery in bilateral eyes was done	Cataract surgery in bilateral eyes was done	Surgery was not performed	A complicated cataract was observed in the left eye, and cataract surgery in the right eye was done	Cataract surgery in the left eye was done	Surgery was not performed
Systemic symptoms	Headache, tinnitus, neck stiffness, hypoacusis, alopecia and poliosis	Headache	Headache, joint pain of upper and lower extremities	Recurrent oral ulcerations, recurrent genital ulcerations and erythema nodosum	Recurrent oral ulcerations, acneiform nodules	Recurrent oral ulcerations, acneiform nodules
Treatment	Oral corticosteroids, oral immunosuppressive agents, corticosteroid eye drops	Oral corticosteroids, oral immunosuppressive agents, corticosteroid eye drops	Oral corticosteroids	Oral corticosteroids, oral immunosuppressive agents, corticosteroid eye drops	Oral corticosteroids, corticosteroid eye drops and adalimumab were administered intravenously for half a year and then withdrawn voluntarily	Oral corticosteroids, corticosteroid eye drops

^a^ OD, right eye.

^b^ OS, left eye.

^c^ KP, keratic precipitates.

^d^ OU, both eyes.

**Figure 1:**
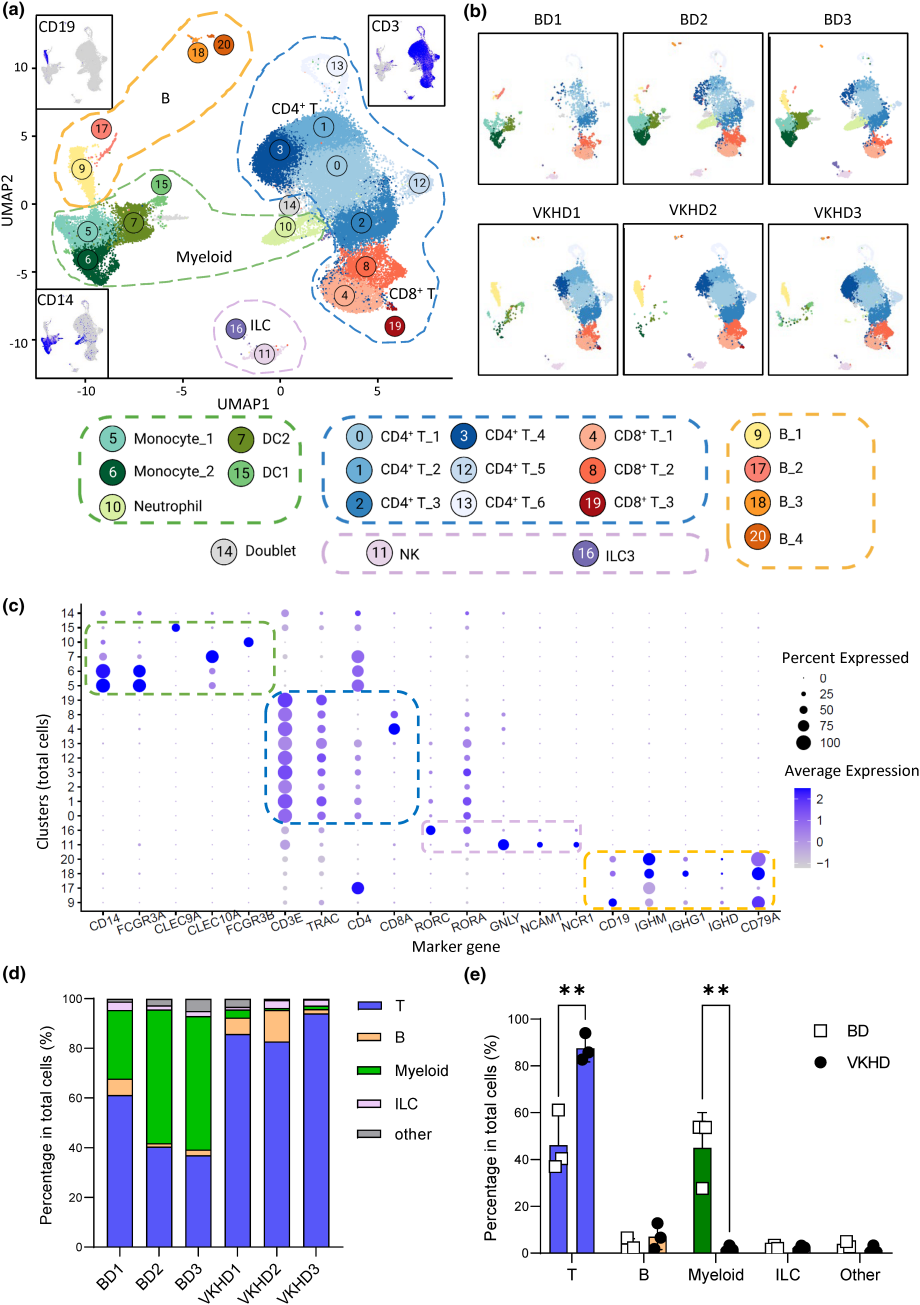
Single‐cell landscape of immune cell infiltration in lesions of autoimmune uveitis in VKHD and BD. **(a)** UMAP plot of unsupervised clustering revealing 21 different cell clusters in aqueous humour analysed by scRNA‐seq (pooled data from six patients, total cell number is 47 078); subgraphs showing the gene expression of *CD3, CD14* and *CD19* in the UMAP plot. **(b)** UMAP visualisation shows single cells and clusters from each patient sample. **(c)** Dot plots show the expression of common marker genes in each cluster for cell type annotation. **(d)** Stacked bar chart of cell type distribution for each patient. **(e)** Bar chart of cell‐type distribution in VKHD and BD samples. Statistical analyses by unpaired *t*‐tests, with significance annotated by **P < 0.01. Data are presented as Mean + SD. BD, Behçet's disease; VKHD, Vogt‐Koyanagi‐Harada disease.

Single cells were extracted from aqueous humour. The total numbers of cells from aqueous humour were between 10^4^ and 10^5^, thus insufficient to conduct high dimensional flow cytometry to investigate various immune markers. We decided to analyse them using single‐cell transcriptomes. Single cells were processed for 5′ gene expression and V(D)J libraries using the Chromium 10× Genomics system. The library preparation, sequencing, read mapping, gene count normalisation and doublet removal were conducted using a standard pipeline.^15^ After doublet removing and integration of data from six samples, a total of 47 048 cells (BD1, 1931; BD2, 7941; BD3, 8097; VKHD1, 6497; VKHD2, 9783; VKHD3, 12 799) underwent the downstream analysis. The single cells were visualised by UMAP in two‐dimensional space. UMAP is a tool to visualise scRNA‐seq data in low dimension for cellular heterogeneity but can also be applied in bulk RNA‐seq to identify sample heterogeneity.^16^ Using a k‐nearest neighbour (KNN)‐based method for clustering, the immune cells in uveitis were classified into 21 clusters (Figure 1a). The clusters were widely distributed in all six samples and did not show any noticeable sample‐specific bias (Figure 1b). We annotated myeloid cells and lymphoid cells that include B cells, T cells and innate lymphoid cells (ILCs) with differential gene expression (DGE) testing among the different clusters (Supplementary figure 1) and checked the DGE lists against manually curated cell type marker genes (Figure 1c).

*CD3E* ^+^ T cells (Clusters 0, 1, 2, 3, 4, 8, 12, 13 and 19) were the dominant immune cell population accounting for 46.22 ± 13.05% (average ± SD, hereinafter) and 87.52 ± 5.84% of total cells from BD and VKHD samples, respectively (Figure 1c–e). In contrast, *CD19* ^+^ *CD79A* ^+^ B cells (cluster 9, 17, 18, 20) only accounted for 3.41 ± 2.78% and 7.04 ± 5.52% of total cells from BD and VKHD samples, respectively (Figure 1c–e). ILCs included *NCAM1* ^+^ *NCR1* ^+^ NK cells (Cluster 11) and *RORC* ^+^ *RORA* ^+^ ILC3(Cluster 16), about 2% each in BD or VKHD samples (Figure 1c–e).

Noticeably, *CD14* ^+^ myeloid cells were mostly presented in the BD samples (45.05 ± 15.02%) and were in very low frequencies in the VKHD samples (1.83 ± 1.30%; Figure 1d, e). Myeloid cells were further clustered into 11 cell types and then annotated by cell type‐specific markers: *FCN1* ^+^ classic monocytes (C0 and C9), *C1Q* ^+^ monocytes (C1 and C2), *CLEC9A* ^+^ cDC1 (C8), *CLEC10A* ^+^ cDC2 (C8), *J‐Chain* ^+^ pDC (C10) and *FCGR3B* ^+^ neutrophil (C3 and C7; Supplementary figure 2a–c). Most myeloid cells, especially monocytes, cDCs and neutrophils, were identified in BD samples (Supplementary figure 2d). Such differences between BD and VKHD samples suggest distinct immune landscapes, in particular T cells and myeloid cells.

### Distinct composition of T cell subsets between BD and VKHD uveitis

**Figure 2:**
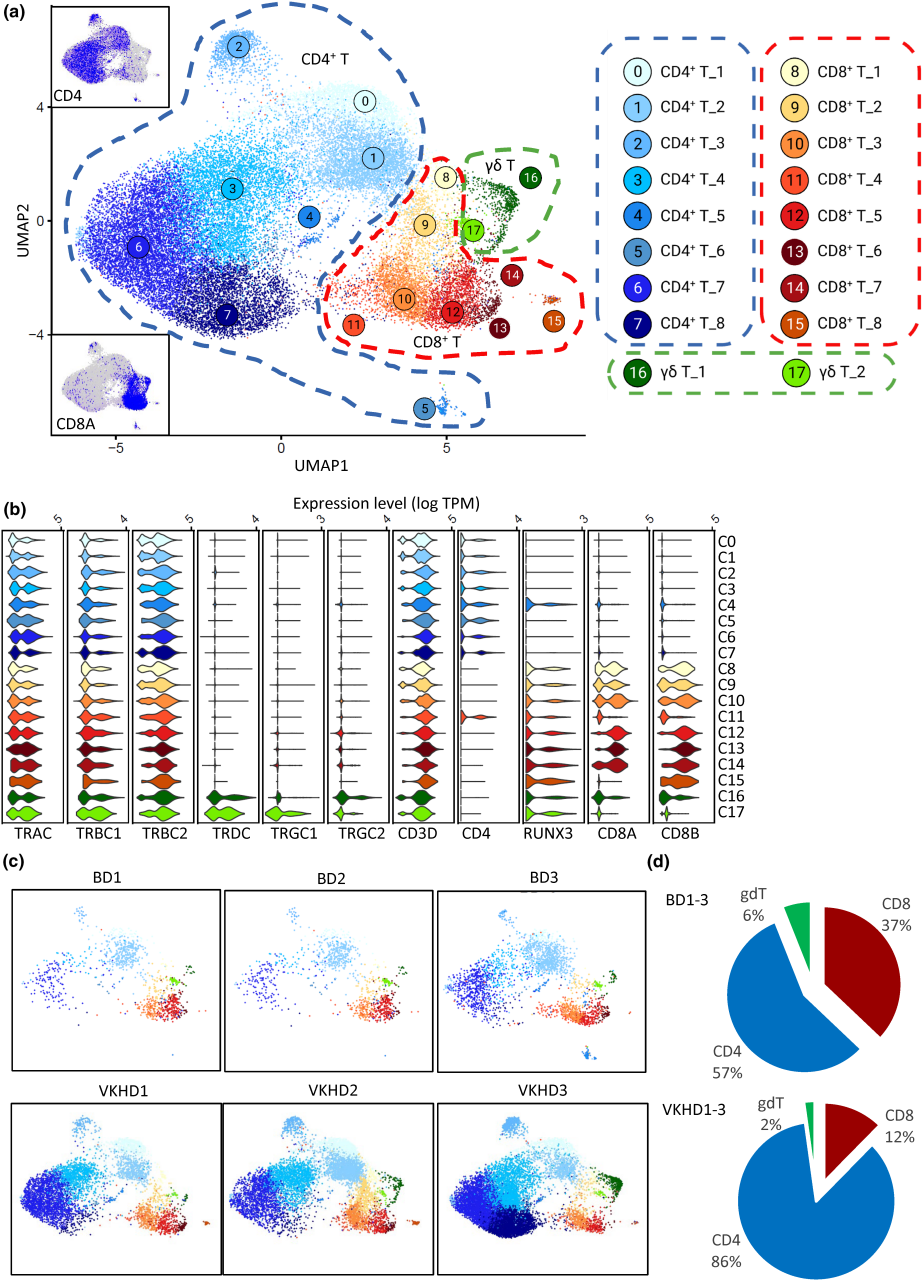
CD4^+^, CD8^+^ and γδ T cells in VKHD and BD uveitis. **(a)** UMAP plot of unsupervised clustering revealing 18 different CD3^+^ T cell clusters sorted in aqueous humour analysed by scRNA‐seq (total cell number is 31 819); subgraphs showing the gene expression of *CD4* and *CD8A* in the UMAP plot. **(b)** Violin plots showing the expression of indicated TCR and co‐receptor genes in each cluster. **(c)** UMAP plots showing single cells and clusters in samples from each patient. **(d)** Pie charts show frequencies of CD4^+^ T cells, CD8^+^ T cells and γδ T cells for BD (top) and VKHD (bottom) samples. BD, Behçet's disease; TCR, T cell receptor; VKHD, Vogt‐Koyanagi‐Harada disease.

We focused on T cells as the major immune cell population in both BD and VKHD samples. The initial clustering of T cells (Clusters 0, 1, 2, 3, 4, 8, 12, 13 and 19 in Figure 1) identified *MKI67* ^+^ proliferating cells that are enriched of G2, M and S cell‐cycle phases (Supplementary figure 3a–c), these cells were removed to improve the analyses of functional cell types.^17^ A total of 18 clusters in T cells were generated (Figure 2a), including eight CD4^+^ T cell clusters (Clusters 0–7) and eight CD8^+^ T cell clusters (Clusters 8–15). Most T cells were predominantly αβ T cells, while we also found a minor population of γδ T cells (Clusters 16, 17; Figure 2b). We did not observe significant enrichment of *Vα24*/*Vβ11* associated with NKT cells or *Vα2*/*Vα7*/*Vβ2/Vβ13* associated with mucosal‐associated invariant T cells in particular subsets^18^, ^19^ (Supplementary figure 3d).

Notably, VKHD samples showed CD4^+^ T cells as the clear majority (VKHD1, 72%; VKHD2, 57%, VKHD3, 85%) with CD4/CD8 ratios 6.89. In contrast, CD4^+^ and CD8^+^ T cell numbers were largely comparable in BD (Figure 2c and d), with the CD4/CD8 ratio of 1.53. This difference suggests a potentially different role of CD4^+^ *vs*. CD8^+^ T cells in driving uveitis in VKHD and BD.

**Figure 3:**
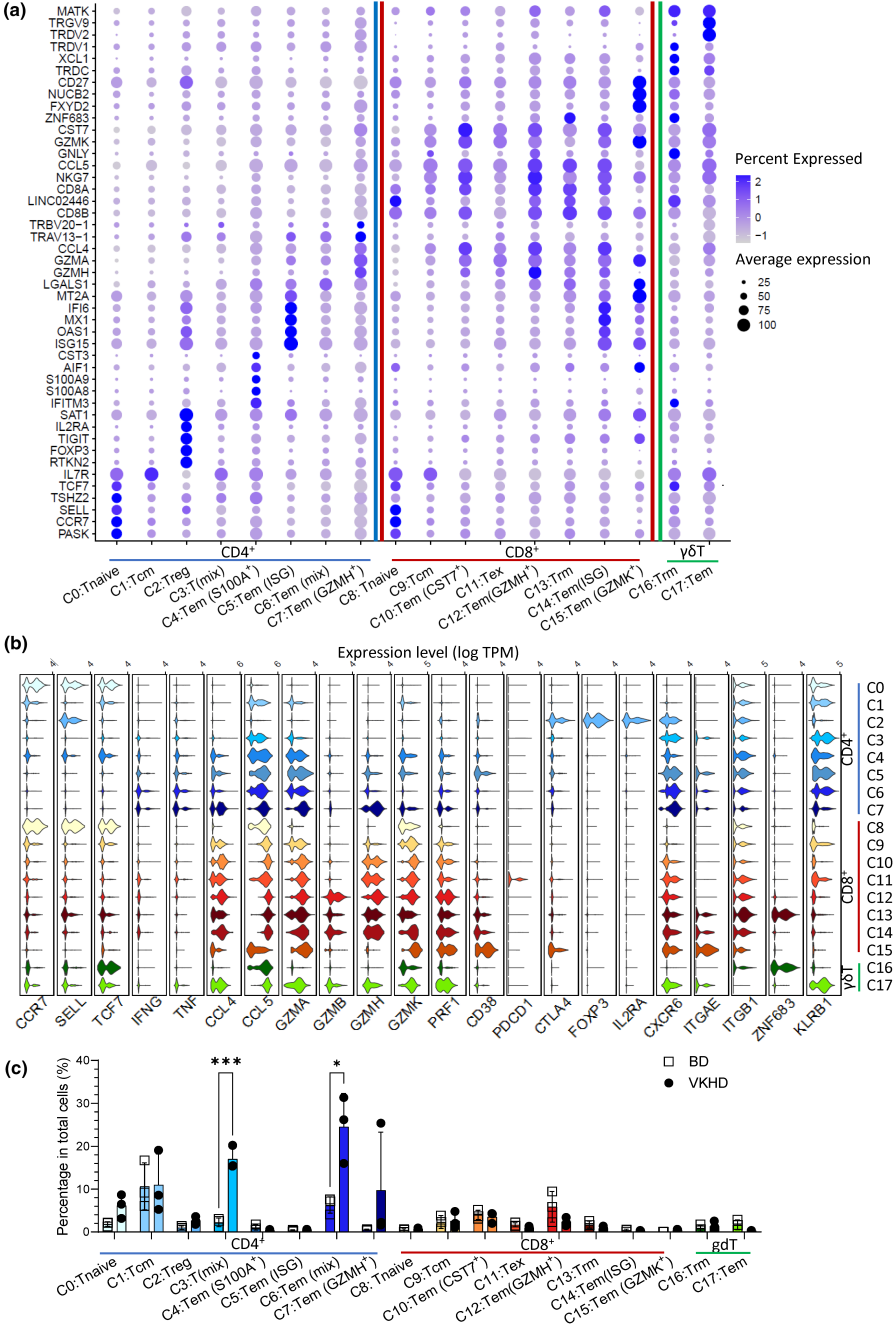
T cell functional subsets differ between VKHD and BD uveitis. **(a)** Dot plots of the top five most upregulated genes for each T cell cluster with a coloured bar dividing CD4 (blue), CD8 (red) and γδ (green) T cells. **(b)** Violin plots of key marker genes for annotating clusters. **(c)** Bar chart of T cell cluster distribution for VKHD and BD. Statistical analyses by unpaired *t*‐tests, with significance annotated by ***P < 0.001 and *P < 0.05. Data are presented as mean + SD. The colours of clusters were matched in UMAP visualisation of T cells in Figure 2. BD, Behçet's disease; VKHD, Vogt‐Koyanagi‐Harada disease.

Using DGE among different clusters, we annotated each cluster's states and functions (Figure 3a and b). Within the CD4^+^ T cells, C0 showed a high expression of *CCR7*, *SELL* (encoding CD62L) and *TCF7* (encoding TCF1) and was annotated as naïve CD4^+^ T cells, whereas C1 was annotated as central memory CD4^+^ T (Tcm) cells. C2 was regulatory T (Treg) cells with the high expression of markers *CTLA4*, *FOXP3* and *IL2RA* (encoding *CD25*). Compared to C1 Tcm cells, C3 downregulated Tcm markers such as CCR7 and SELL but yet to express effector molecules such as *IFNG, TNF, CCL4, CCL5* or granzymes, so it is more likely to be a mix or transition of memory and effector T cells. C4 was effector memory T (Tem) cells with the expression of S100 protein genes (*S100A8, S100A9*), which can induce inflammatory cells to secrete a variety of cytokines to maintain and exacerbate inflammation.^20^ C5 and C6 were also annotated as Tem, but C5 had a high expression of IFN‐I signalling correlated genes (*IFI6, MX1, OAS1, ISG15*). Notably, C7 was marked by the highest expression of *GZMK*. *GZMK* ^+^ Tem C7 was highly pro‐inflammatory, as indicated by the highest levels of *IFNG, TNF*, CCL and granzyme molecules and *PRF1* (Figure 3b).

Within the CD8^+^ T cells, C8 was a naive CD8 population with the highest expression of *CCR7*, *SELL* and *TCF7* (Figure 3a and b). C9 was CD8^+^ Tcm cells. C10, C12, C14 and C15 were CD8^+^ Tem clusters but differentially marked by signature genes for effector molecules (C10 – *CST7*, C12 – *GZMH*, C14 – ISG signature as C5, C15 – *GZMK*; Figure 3a). C11 upregulated *PDCD1* and *CTLA4*, but downregulated effector molecules, thus being annotated as exhausted CD8^+^ T (Tex) cells (Figure 3b). C13 was a resident memory CD8^+^ T (Trm) subset because of the upregulation of *ZNF683* (encode Hobit), *ITGAE* (encode CD103) and *ITGB1* (encode CD49a) while the levels of effector molecules were low (Figure 3b).

Within the two clusters of γδ T cells, C16 was the Trm subset with high expression of *ZNF683* but low effector molecules while C17 was the Tem subset with much higher expression of CCL and granzyme molecules than C16 **(**Figure 3a and b **).**

After annotating T cell functional clusters, we compared the frequencies of CD4^+^ and CD8^+^ T cell clusters between VKHD and BD. The higher CD4/CD8 ratios in VKHD than BD (Figure 2d) were found primarily driven by the expansion of certain CD4^+^ clusters in VKHD. Compared to BD, the major expanded CD4^+^ T cell clusters in VKHD were C3 – T (MIX), C6 – Tem (MIX) and C7 – Tem (*GZMH* ^+^), whereas naïve (C0), Tcm (C1) and Treg (C2) showed little or minor changes (Figure 3c). Naïve (C8) and Tcm (C9) CD8^+^ clusters were also comparable between VKHD and BD. However, compared to VKHD, BD showed a trend of increase in C12 – Tem (*GZMH* ^+^), although these did not reach statistical significance (Figure 3c).

**Figure 4:**
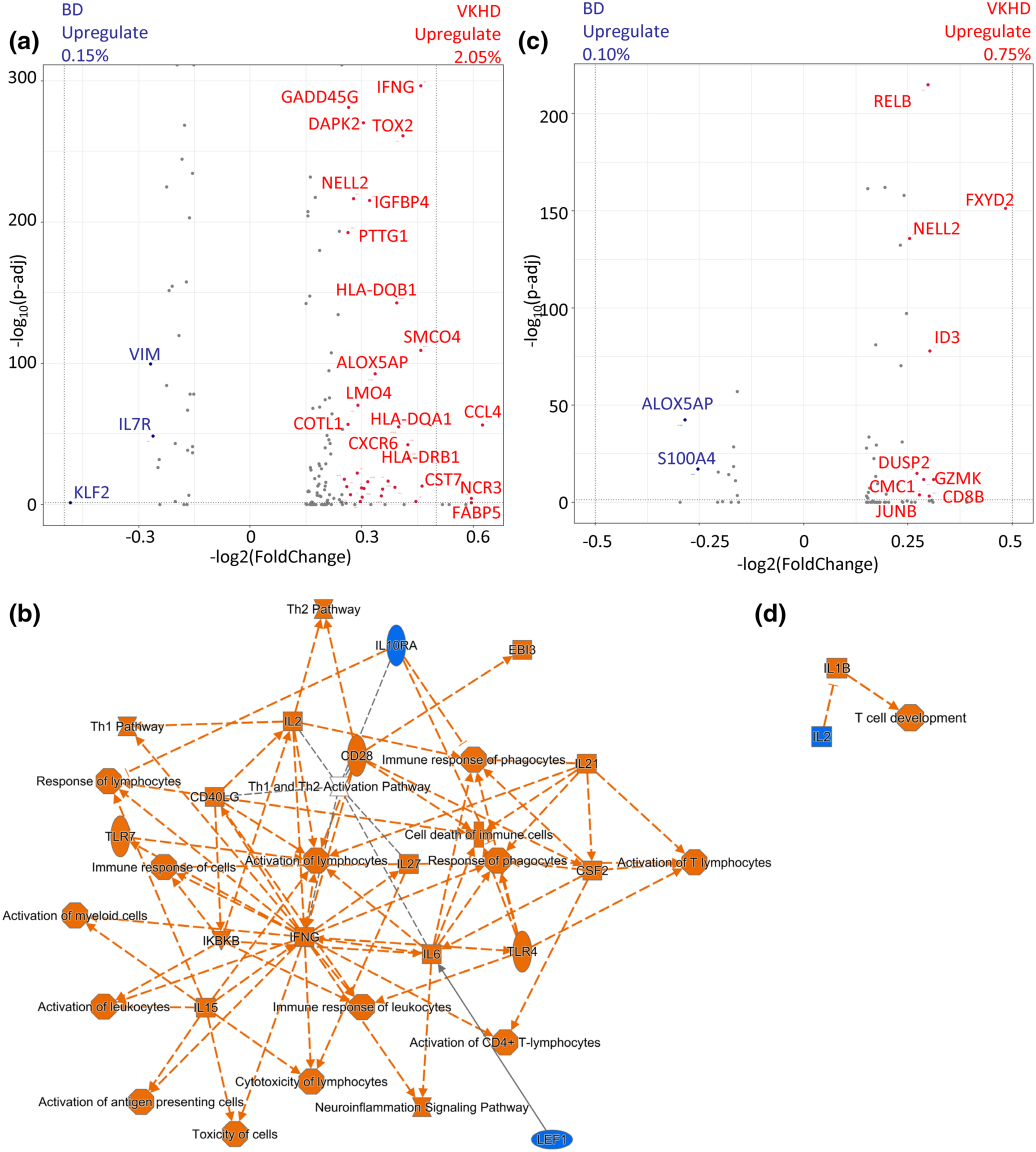
DGE and functional pathways between VKHD and BD. **(a, b)** Volcano plots of DGE between VKHD (right‐hand side, red) and BD (right‐hand side, red) in CD4^+^ **(a)** and CD8^+^ **(b)** T cells. Genes were annotated significantly with *P* < 1 × 10^−5^ and an absolute number of fold change > log_2_0.25. For all volcano plots, *P*‐values were calculated using a Wilcoxon rank‐sum test. **(c, d)** IPA graphical summary indicating activated (orange) and inactivated (blue) genes or pathways comparing VKHD with BD in CD4^+^ **(c)** and CD8^+^ T cells **(d)**. Solid arrows indicate direct action, and dashed lines indicate indirect action. BD, Behçet's disease; DGE, differentially expressed genes; IPA, ingenuity pathway analysis; VKHD, Vogt‐Koyanagi‐Harada disease.

We also compared gene expression in CD4^+^ or CD8^+^ T cells between VKHD and BD. In line with the bias of CD4^+^ T cells in VKHD towards Tem clusters (Figure 2c), they also upregulated the expression of Th1 effector molecules than in BD counterparts, as evidenced by *IFNG, CCL4* and *GADD45G* ^21^ (Figure 4a). Ingenuity pathway analysis (IPA) revealed that upregulated gene expression in VKHD was associated with pathways for T cell activation and *IFNG* (Figure 4b). There were few differential expression genes between VHKD and BD CD8^+^ T cells (Figure 4c). As a consequence, there was no clear indication of pathway difference by IPA (Figure 4d). We noticed that CD8^+^ T cells in VHKD, compared to BD, showed higher expression of *ID3* which is associated with CD8^+^ T cell memory differentiation^22^ and NELL2 which is highly expressed by naïve CD8^+^ T cells^23^ (Figure 4c). This suggests that CD8^+^ T cells in VHKD were in general more resting and less activated compared to those in BD. Altogether, the different CD4^+^ and CD8^+^ T cell composition in uveitis tissue infiltrates are largely driven by effector memory populations, suggesting the effector function of CD4^+^ and CD8^+^ T cells preferentially activated in VKHD and BD respectively.

### Divergent T cell clonal expansion in BD and VKHD uveitis

T cell proliferation and functional differentiation are usually driven by antigen recognition through specific TCR. We then analysed TCR sequences of T cells in all six patient samples, with an emphasis on clonality by quantifying the frequencies of T cells with a common CDR3 sequence. According to the percentages of the T cell numbers of each T cell clone in the total T cells from a given sample, clones were classified into three groups: large (> 2% of total T cells), medium (0.5–2%) and small (< 0.5%) clonotypes.

**Figure 5:**
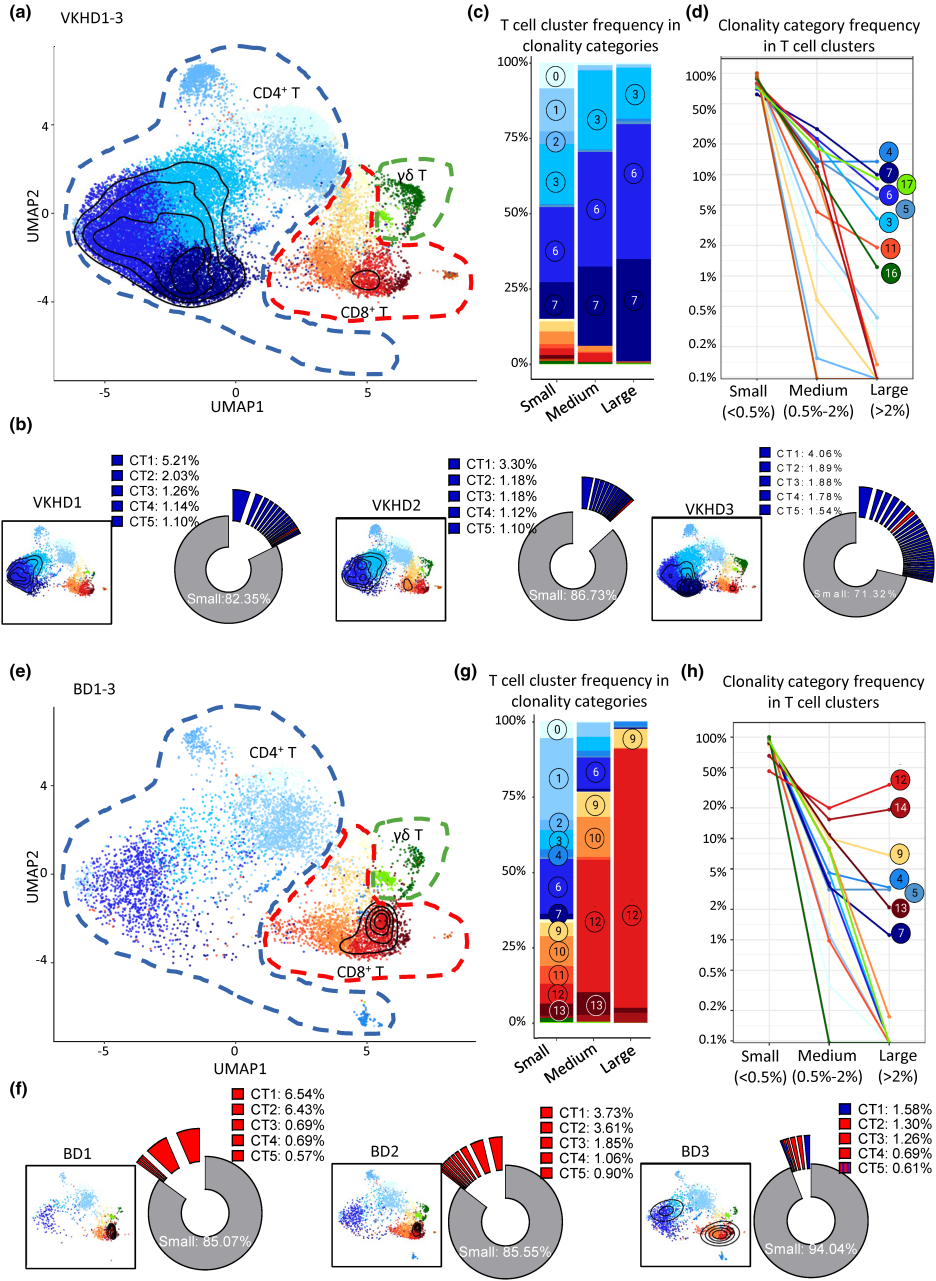
Divergent T cell clonal expansion in BD and VKHD uveitis. **(a, e)** Density contour plots overlayed with UMAP plots showing the distribution of T cells in large and medium clonotypes (> 0.5% of total T cells). Pooled samples in VKHD **(a)** and BD **(e)**. **(b, f)** Density contour plots overlayed with UMAP plots show the distribution of T cells in large and medium clonotypes in individual samples (left panels), and donut explosion plots show large and medium clonotypes (> 0.5% T cells) with the frequencies of top five expanded clonotypes indicated (right panels). Individual samples in VKHD **(b)** and BD **(f). (c, g)** Stacked bar charts for the frequencies of T cell clusters in clonality categories in VKHD **(c)** and BD **(g)**. **(d, h)** Connected dot plots show the proportions of clonality category in each T cell cluster in VKHD **(d)** and BD **(h).** Large clonotypes > 2% of all T cells with TCR sequencing data detected from the same patient. Medium: 0.5–2% of all T cells with TCR sequencing data detected from the same patient. Small: < 0.5% of all T cells with TCR sequencing data detected from the same patient. BD, Behçet's disease; TCR, T cell receptor; VKHD, Vogt‐Koyanagi‐Harada disease.

There were no identical CDR3 sequences of large or medium clonotypes from different samples (Supplementary table 1). To visualise the distribution of expanded T cell clones in either disease, we generated topographic maps for large and medium clonotypes in the UMAP plots of T cells. In VKHD, major T cell clones (large and medium clonotypes > 0.5% of total T cells) were predominantly CD4^+^ T cells and had a minor contribution from CD8^+^ T cells. They were mostly in Tem clusters (C6 and C7) and some in the mixed cluster (C3) CD4^+^ T cells (Figure 5a). Selective clonal expansion in CD4^+^ T cells was consistent in all three VKHD samples (Figure 5b). Mixed (C6) and *GZMH* ^+^ (C7) CD4^+^ Tem cells accounted for about 80% of large clonotypes and about 70% of medium clonotypes (Figure 5c). Clonal expansion occurred mostly in C6 and C7 CD4^+^ Tem cells could not be simply explained by the large numbers of cells in C6 and C7 clusters. Indeed, C6 and C7 CD4^+^ Tem cells only accounted for less than 40% of small clonotypes (Figure 5c). Active clonal expansion in Tem cells was also confirmed by quantifying the frequencies of small, medium and large clones in each T cell cluster, which demonstrated that C6 and C7 CD4^+^ Tem T cells, along with C5 S100A^+^ Tem CD4^+^ T cells and C17 γδ Tem cells, were ranked the highest for the frequencies of large clonotype cells (Figure 5d). These results suggest a key role of antigens, possibly autoantigens, in driving the expansion of pro‐inflammatory CD4^+^ Tem cells and γδ Tem cells in VKHD.

In contrast, T cell clonal expansions were almost exclusively in CD8^+^ T cells in BD (Figure 5e). Again, selective clonal expansion in CD8^+^ T cells was consistent in all three BD samples (Figure 5f). Strikingly, C12 *GZMH* ^+^ CD8^+^ Tem cells alone accounted for almost 90% of large clonotypes and almost 50% of medium clonotypes (Figure 5g). By quantifying the frequencies of small, medium and large clones in each T‐cell clusters, C12 *GZMH* ^+^ and C14 ISG CD8^+^ Tem cells were ranked at the top for the frequencies of large clonotype cells (Figure 5h). These results suggest that, in contrast to the pathogenic role of clonally expanded CD4^+^ T cells in VKHD, antigens, or autoantigens, preferentially drive the expansion of pro‐inflammatory CD8^+^ Tem cells in BD.

Genetic studies have reported that VKHD is associated with MHC class II‐specific allele HLA‐DR4/HLA‐DRB1*04, whereas BD is associated with MHC class I‐specific allele HLA‐B*51.^24^, ^25^ We performed HLA analyses on single‐cell sequencing data from six autoimmune uveitis patients (Supplementary figure 4a). HLA‐B*51 was found in one in three BD patients, whereas HLA‐DR4 (HLA‐DRB1*04) was found in two of three VKHD patients and one of three BD patients (Supplementary figure 4b). Our data therefore suggest that the susceptible MHC class I allele HLA‐B*51 may promote (auto)antigen presentation but is not necessary to induce selective expansion of pro‐inflammatory CD8^+^ Tem cells in BD. Similarly, the susceptible MHC class II allele HLA‐DR4/HLA‐DRB1*04 can be often found in VHKD patients with expanded pro‐inflammatory CD4^+^ Tem cells in VHKD but other MHC class II alleles can also achieve this.

**Figure 6:**
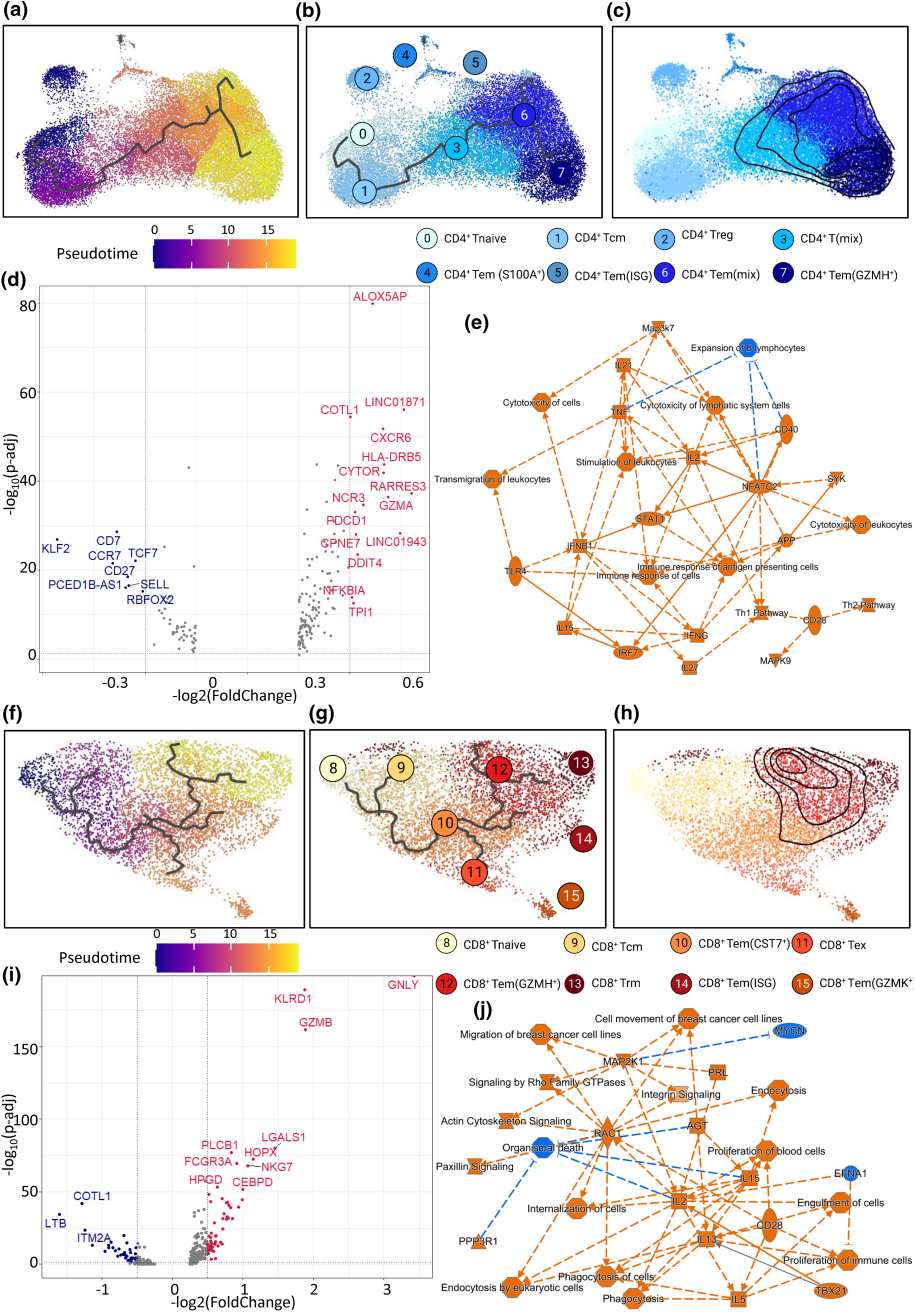
Differentiation trajectories and functional features of clonally expanded T cells in BD and VKHD uveitis. **(a–c)** UMAP plots of CD4^+^ T cells in pooled VKHD and BD samples showing the trajectory overlayed with pseudotime **(a)** or clusters **(b)** or showing the overlayed density contour plots for the distribution of CD4^+^ T cells in large and medium clonotypes (> 0.5% of total T cells in individual samples) **(c)**. **(d)** Volcano plot shows DGE between CD4^+^ T cells in large clonotypes (> 2%) and other CD4^+^ T cells. **(e)** IPA plot showing activated (orange) and inactivated (blue) genes or pathways based on DGE in **(d)**. Solid arrows indicate direct actions, and dashed lines indicate indirect actions. **(f–h)** UMAP plots of CD8^+^ T cells in pooled VKHD and BD samples show the trajectory overlayed with pseudotime **(f)** or clusters **(g)** or show the overlayed density contour plots for the distribution of CD8^+^ T cells in large and medium clonotypes (> 0.5% of total T cells in individual samples) **(h)**. **(i)** Volcano plot shows DGE between CD8^+^ T cells in large clonotypes (> 2%) and other CD4^+^ T cells. **(j)** IPA plot shows activated (orange) and inactivated (blue) genes or pathways based on DGE in **i**. Solid arrows indicate direct actions, and dashed lines indicate indirect actions. *P*‐values in volcano plots were calculated using Wilcoxon rank sum tests. Annotated genes for significant *P*‐values < 1 × 10^−5^ and fold changes > log_2_0.5. BD, Behçet's disease; DGE, differentially expressed genes; IPA, ingenuity pathway analysis; VKHD, Vogt‐Koyanagi‐Harada disease.

After identifying the clonal expansion of pro‐inflammatory CD4^+^ and CD8^+^ Tem cells in VKHD and BD, respectively, we next performed trajectory analyses to understand the generation of pathogenic T cell clones. We first used *monocle3* to generate the trajectory of CD4^+^ T cells based on pseudotime (Figure 6a). C0 naïve CD4^+^ T cells were chosen as the starting point of the differentiation (Figure 6b). The differentiation of CD4^+^ T cells progressed from C0 (naïve) to C1 (Tcm), and then to C6 (Tem) *via* the transition of mix effector and memory C3, eventually reaching C7 (*GZMH* ^+^ Tem; Figure 6b). Such trajectory analysis supported the notion that the continuous antigen exposure drove the expansion of medium and large colonotypes in C6 and C7 Tem (Figure 6c). We then focused on large CD4^+^ clonotypes and conducted their DGE compared to other CD4^+^ T cells. CD4^+^ T cells with large clonotypes strongly downregulated memory markers *CCR7, SELL, KLF2* and *TCF7*, suggesting effector differentiation (Figure 6d). IPA based on DGE predicts such cells are pro‐inflammatory Th1 cells (Figure 6e, Supplementary figure 5a). Such analyses are similar to the pathway analyses in CD4^+^ T cells between VKHD *vs*. BD. This again suggests that antigen‐induced expansion of pro‐inflammatory Th1 cells is the feature of VKHD compared to BD.

We also generated the trajectory of CD8^+^ T cells based on pseudotime (Figure 6f). C8‐naïve CD8^+^ T cells were chosen as the starting point of the differentiation (Figure 6g). The differentiation of CD8^+^ T cells progressed from C8 (naïve) to C10 (*CST7* ^+^ Tem), with a branch out to C9 (Tcm). C10 then differentiated into C11 (Tex) or C12 (*GZMH* ^+^ Tem; Figure 6g). Similar to the CD4^+^ T cell trajectory, the expansion of medium and large clonotypes in the C12 CD8^+^ Tem likely results from antigen stimulation (Figure 6h). CD8^+^ T cells with large clonotypes strongly upregulated cytotoxic markers *GZMB, GNLY* and *NKG7* (Figure 6i). IPA based on DGE revealed showed pathway activation for not only cytotoxicity but also cytoskeleton remodelling and cellular adhesion (Figure 6j, Supplementary figure 5b). Our results suggest that, although there are clonal expansions of pro‐inflammatory Tem cells in both VHKD and BD, the two types of autoimmune uveitis differ in the type of pathogenic T cells, with Th1 cells in VKHD and cytotoxic CD8^+^ T cells in BD.

## Discussion

Ocular immune privilege has been supported by multiple facets of evidence.^26^ The prominent presence of immune cells, especially T cell infiltration in autoimmune uveitis in BD and VKHD suggests that T cells may play a significant role in the immunopathogenesis, which is also supported by results from mouse models of experimental autoimmune uveitis (EAU) and clinical benefits observed from T cell‐targeting therapies.^27^ However, the observations of immune dysregulation in different human studies were not always consistent, not only due to the purposes of study design and methodologies in data collection but also reflecting the heterogeneity in immunopathogenesis in autoimmune uveitis. Systems immunology approaches have been taken to stratify uveitis patients, such as by peripheral blood transcriptomic analyses,^28^ but immune signatures in ocular tissues cannot be fully recapitulated in peripheral blood. The latest advancement in scRNA‐seq technology allows us to directly investigate immune responses in the ocular microenvironment, thus providing unprecedented opportunities to understand both immunopathogenesis and the heterogeneity among patients.^29^, ^30^ In addition, scTCR‐seq can efficiently identify clonal T cells and pave the way to interrogate the question whether the function of T cells is driven by antigen stimulation.

Our scRNA‐seq of cells in aqueous humour from autoimmune uveitis patients confirmed a dominant presence and pro‐inflammatory phenotypes of T cells. More importantly, the analyses revealed that T cell phenotypes in VKHD‐associated uveitis were different from those in BD‐associated uveitis. Uveitis in VKHD patients showed the dominant role of clonally expanded Th1‐like effector CD4^+^ T cells expressing *IFNG* and *TNF*. In contrast, uveitis in the BD patient had few of these effector CD4^+^ T cells but was characterised by clonally expanded effector CD8^+^ T cells expressing *CCL5, GZMA, GZMB* and *PRF1*.

The differential contribution of CD4^+^ and CD8^+^ T cells in autoimmune uveitis in VKHD and BD, respectively, is also supported by the analyses of T cell clonality. After integrating scRNA‐seq and scTCR‐seq data, we saw a clear clonal expansion in effector memory CD4^+^ T cells (the *GZMH* ^+^ CD4^+^ Tem cluster, C7) with a Th1‐like phenotype in VKHD but not BD. Different from previous studies using PBMCs in VKHD or a mouse model for EAU that reported a role of Th17 cells, we did not observe strong Th17 signatures in immune infiltrates in humour aqueous of VKHD patients.^14^ This could be explained by the Th17 signature detected in the blood of VKHD patients might be more relevant to other tissue complications rather than uveitis or by the conceivable difference between immune signatures in ocular tissues and peripheral blood. After integrating scRNA‐seq and scTCR‐seq data, we saw a clear clonal expansion in effector memory CD4^+^ T cells (the *GZMH* ^+^ CD4^+^ Tem cluster, C7) with a Th1‐like phenotype in VKHD but not BD. Different from previous studies using PBMCs in VKHD or a mouse model for EAU that reported a role of Th17 cells, we did not observe strong Th17 signatures in immune infiltrates in humour aqueous of VKHD patients.^14^ This could be explained by the Th17 signature detected in the blood of VKHD patients might be more relevant to other tissue complications rather than uveitis or by the conceivable difference between immune signatures in ocular tissues and peripheral blood. Follicular helper T (Tfh) cells have a profound role in supporting self‐reactive humoral immunity, particularly autoantibody production, thus promoting autoimmune immune diseases.^31^, ^32^ However, we did not detect Tfh‐related gene signatures, such as the Tfh master transcription factor BCL6.^33^ This suggests that self‐reactive humoral immunity is not essential in uveitis associated with VKHD and BD. In contrast, it was effector memory CD8^+^ T cells (the *GZMH* ^+^ CD8^+^ Tem cluster, C12) with a cytotoxic phenotype that expanded in the ocular tissues in BD but not VKHD uveitis. The results strongly suggest the distinctive roles of CD4^+^ and CD8^+^ T cells in the pathogenesis of autoimmune uveitis between VKHD and BD. Such difference is consistent in all six autoimmune uveitis patients including three VKHD and three BD. Similar to two recent scRNA‐seq studies of aqueous immune cells from four to five samples,^29^, ^30^ a major limitation of our study was the sample size. Future studies are required to use a larger cohort to validate the distinctive roles of CD4^+^ or CD8^+^ T cells in autoimmune uveitis in VKHD and BD.

The notion that CD4^+^ and CD8^+^ T cells play distinctive roles in VKHD and BD respectively is supported by genetic studies including genome‐wide association studies. In multiple populations, BD was found associated with MHC class I‐specific allele HLA‐B*51, whereas VKHD was associated with MHC class II‐specific alleles *HLA‐DR4/HLA‐DRB1*.^24^, ^25^ In our cohort, HLA‐B*51 was found in one in three BD patients whereas *HLA‐DR4* (HLA‐DRB1*04) was found in two of three VKHD patients and one of three BD patients. Therefore, other MHC class I alleles other than HLA‐B*51 may also promote (auto)antigen presentation to induce pathogenic pro‐inflammatory CD8^+^ Tem clonal expansion. Interestingly, even with the susceptible MHC class II allele HLA‐DR4/HLA‐DRB1*04, one BD patient did not induce pathogenic pro‐inflammatory CD8^+^ Tem clonal expansion.

The important question following our study is what (auto)antigens drive the clonal expansion of pathogenic proinflammatory CD4^+^ and CD8^+^ Tem cells in VKHD and BD respectively. We used the TCR sequences in the ‘large’ clonotypes (> 2%) from each patient to search VDJDB, a public database for TCR sequences and their corresponding antigens based on published and experimental data.^34^ We could identify that the CDR3 β chain sequences from certain clonotypes were identical or very similar (differing by one amino acid) in the database, but their CDR3 α chain sequences significantly differed from the relevant CDR3 α chain sequences in the database (data not shown). Therefore, there were no CDR3 sequences from highly expanded T cell clones in autoimmune uveitis that perfectly matched with reported TCR sequences, which are mostly for the recognition of microbes, such as viruses. Although it is possible that expanded CD4^+^ or CD8^+^ T cell clones in autoimmune uveitis recognise microbial epitopes not included in the database, we favour the hypothesis that these expanded T cell clones are driven by the recognition of ocular autoantigens. If such (auto)antigens can be identified, the information can be used in the diagnosis and also design therapies to specifically induce the tolerance to these antigens^35^ and, thus, suppress the generation of pro‐inflammatory T cells.

In both VKHD and BD, Treg cells (C2) only accounted for a small fraction of T cells (BD:2.43% and VKHD:3.04%). A striking observation was that Treg cells (C2) underwent the least clonal expansion in both VKHD and BD, suggesting that insufficient expansion of Treg cells might posit in the root of losing immune tolerance in autoimmune uveitis. Our previous clinical trials of low‐dose IL‐2 therapy in systemic lupus erythematosus and Sjogren's syndrome have demonstrated that it could enhance antigen‐independent expansions of Treg cells,^36^, ^37^, ^38^ thus providing a strategy to improve the poor clonal expansion of Treg cells in autoimmune uveitis. However, IPA analyses also suggest that IL‐2 could be involved in the generation of pro‐inflammatory CD4^+^ Tem and CD8^+^ Tem cells. Excessive IL‐2 signalling could drive CD8^+^ T cells‐mediated tissue immunopathology, as seen in viral infections.^39^, ^40^ Future studies are required to further study how IL‐2 is involved in the modulation of the balance of Treg cells and pathogenic pro‐inflammatory Tem cells in autoimmune uveitis.

Besides T cell phenotype and clonality, another major difference in autoimmune uveitis between VKHD and BD was the significant infiltration of myeloid cells, including monocytes, neutrophils and dendritic cells, in ocular tissues of BD but not VKHD, which accounted for 25–50% of total immune cell infiltrates. In agreement with a previous study that reported the expansion of C1Q^hi^ monocytes in PBMCs of BD,^10^ we also identified two C1Q^+^ monocyte clusters in BD. However, intermediate monocyte, cDC2 and neutrophil clusters were also highly expanded in BD than VKHD. Thus, it is conceivable that these myeloid clusters may work synergistically to produce inflammatory cytokines and chemokines and to attract and present antigens to pathogenic T cells. We observed very low levels of B cell infiltration in ocular tissues. Although clinical evidence supports the efficacy of wB cell depletion therapy with rituximab, an anti‐CD20 antibody, against refractory chronic recurrent VKHD,^41^ the benefits might primarily rely on its actions on the peripheral B cell populations rather than ocular tissues.

Taken together, our study provides a landscape view of immune infiltration in ocular tissues in autoimmune uveitis and reveals major differences between VKHD and BD. Such discoveries emphasise the focus on myeloid cells and cytotoxic CD8^+^ Tem cells in BD and pro‐inflammatory Th1‐like CD4^+^ Tem cells in VKHD in understanding disease immunopathogenesis and developing precision therapies.

## Methods

### Human subjects

This study was performed at Beijing Chaoyang Hospital, Capital Medical University, Beijing, China. The ethics approval was obtained from the Ethics Committee of Beijing Chaoyang Hospital (Approval Number: 2018‐4‐3‐3). Written informed consents were obtained from all patients. Three VKHD patients and three BD patients (aged from 8 to 39 years old) were enrolled at the Ophthalmology Department of Beijing Chaoyang Hospital affiliated with Capital Medical University. All patients had bilateral or unilateral vision loss and had cataract surgeries.

### Single‐cell collection from aqueous humour

Aqueous humour of about 100 μL was taken from individual patients after an anterior chamber paracentesis (ACP). The ACP was carried out by using a 30‐gauge needle, with a 1‐mL insulin syringe, *via* the temporal limbal approach and a rolling technique. Aqueous humour was aspirated and immediately aliquoted into a microfuge tube. All the aqueous humour samples were centrifuged at 800 *g* for 15 min at 4°C. No red blood cells were observed in aqueous humour, so we did not use red blood cell lysis buffer to remove red blood cells. The pellets were washed twice with cold phosphate‐buffered saline (PBS). Cells were counted by counting Star (Counstar Rigel S2, Shanghai Ruiyu Biotechnology, China).

### Single‐cell RNA sequencing

10× Genomics Platform was used for single‐cell RNA sequencing. Single‐cell suspension with gel beads (containing the pre‐made 10× primers) and Master Mix together formed Gel Bead‐In‐EMulsions (GEMs). Cleavage and reverse transcription were performed in GEMs. After cDNA amplification was completed, we performed a quality inspection and finally constructed the 5′ libraries for transcriptomes and TCR sequencing. The Illumina Novaseq 6000 sequencing platform was used for sequencing, obtaining sequencing data and performing subsequent data analysis.

### Data processing

The samples were sequenced in high‐throughput using the 10× single‐cell application and the double‐end sequencing mode of the Illumina sequencing platform, and the pre‐processed data were analysed for quality control using *FastQC* software. *Cell Ranger* (3.0.1) was used to compare the Read2 sequences to the *Ensembl* reference genome sequence (GRCh38 for humans) using STAR on the raw data. The software distinguished between Barcode sequence markers of cells and UMI markers of different mRNA molecules within cells by a cell identification algorithm and counted the number of cells detected, the number of genes and the cell and gene expression matrix after filtering and filtering. *scHLAcount* (0.1.0) extracted HLA allele sequences and relevant read from *Cell Ranger* BAM file, enabling specific analysis of HLA class I and class II alleles in single‐cell gene expression data.

Single‐cell RNA‐seq and TCR‐seq data were both analysed in the environment of *R‐4.2.2. Seurat* ^42^ was used for scRNA‐seq data processing. For scRNA‐seq data, each sample data was filtered by *Doubletfinder* R package to remove doublets^43^ and 7.5% was removed as recommendation. Subsequently, all filtered cells from six patient samples were integrated using functions in *Seurat* package to eliminate the batch effect. *FindIntegrationAnchors* was used to find a set of anchor features that helps integration. CCA was used for dimensional reduction of datasets, and L2 normalisation was performed. The selected ‘anchor features’ can be used to unify the data set into a single reference. Then we used *IntegrateData* function in Seurat to engage data integration using anchors provided, and 30 dimensions were used in the anchor weighting procedure. After integration, a new assay ‘integrated’ was used for most of the downstream analysis.

The data were normalised to a scale of 10 k by *NormalizeData* and then scaled *via ScaleData* in *Seurat. The scaled data* were used for generalising further downstream analysis and visualisation. We used runPCA to reduce the feature dimension of scRNA‐seq data from 2000 (anchor features) to 50, which was useful in visualisation. *Findneighbors* was a function of calculating the KNNs for each cell in the dataset and creating the nearest neighbour graph using the overlaps between KNNs. The graph was used for running unsupervised clustering using *FindClusters* in *Seurat*.

After sorting from all cells using CD3 markers, T cells from six samples were initialized for the first time. Based on initial clustering results, we used *CellCycleScoring* to calculate cell cycle scores of T cells and used *Featureplot* to show cell state marker ki67 (MKI67) expression. Based on the results, we removed high proliferate T cell clusters in initial clustering results. Meanwhile, CD8 cells were subclustered using *FindSubCluster* due to its internal heterogeneity. Finally, all T cell clusters were renamed and sorted based on cell type and its differentiation state.

### Visualisation

scRNA‐seq data were visualised using the dimensionality reduction method of UMAP (Uniform Manifold Approximation and Projection). Stacked violin plots were made by a modified function of *Vlnplot* in *Seurat*. Heat maps showing the top five highest differential expressed genes in each cluster were made by *DoHeatmap* function. DGE analyses were performed using *FindMarkers* and *FindAllMarkers* in *Seurat* with the parameter of adjusted *P*‐value threshold < 0.05. Trajectory analysis was performed by *learn_graph, order_cells* and *plot_cells* in the *Monocle3* R package and based on the UMAP reduction method.^44^

### Pathway analysis

Qiagen IPA was performed in this study. The differential expressed genes were uploaded as input as well as their *P*‐value, log fold‐change and adjusted *P*‐value. IPA automatically compare gene list against the public data sets and previous experiments and match gene list to the pathways or specific disease. IPA helps generate a summary figure showing the activated and inactivated genes and pathways.

### Single‐cell TCR clonotype analysis

V(D)J sequencing data were analysed by *CellRanger*. The *CellRanger* VDJ function was used for Contig assembly, cross‐cell Consensus sequence and CDR3 Clonotype typing of TCR gene V(D)J region sequences of a single cell. *Seurat* and scRepertoire^45^ were used to add VDJ sequencing data as additional data to merge with single‐cell sequencing data. TCR clonotypes were defined by identical CDR3 amino acid sequences for both α and β chains. Clonality categories were calculated by dividing cells in the clonotype by all T cells with TCR sequencing data detected from the same patient. Clonotypes were classified as small (< 0.5%), medium (0.5–2%) and large (> 2%) clonotypes. For each patient, pie charts were used to show the top five largest clonotypes and all large and medium clonotype proportions. We used blue colour to show CD4^+^ clonotypes and red for CD8^+^ clonotypes, whereas red and blue crossed lines showed rare clonotypes which include both CD4^+^ and CD8^+^ T cells.

### Statistical analyses

Bar charts and histograms were generated by Prism V9 and Microsoft Excel. Comparisons were performed by unpaired *t*‐tests with *P*‐values less than 0.05 as statistical significance, *P < 0.05, **P < 0.01, ***P < 0.001.

## Author Contributions

**Hao Kang:** Data curation; formal analysis; project administration. **Hongjian Sun:** Data curation; formal analysis; investigation; project administration; writing – original draft; writing – review and editing. **Yang Yang:** Methodology; supervision. **Zewen K Tuong:** Methodology; supervision. **Minglei Shu:** Funding acquisition; resources; supervision. **Yunbo Wei:** Project administration; resources. **Yu Zhang:** Project administration; supervision. **Yong Tao:** Conceptualization; funding acquisition; investigation; project administration; resources; supervision; writing – original draft; writing – review and editing. **Di Yu:** Conceptualization; formal analysis; investigation; supervision; writing – original draft; writing – review and editing.

## Conflict of interest

The authors declare no competing interest.

## Supporting information


Supplementary figures 1‐5

Supplementary table 1

## Data Availability

The data sets during this study are available at the Science DataBase: https://www.scidb.cn/s/bAF7fe. Codes are available at https://github.com/HongjianSun/Uveitis‐project.
