# Supplementary material for: Autoimmune uveitis in Behçet's disease and Vogt‐Koyanagi‐Harada disease differ in tissue immune infiltration and T cell clonality
Source: Clin Transl Immunology. 2023 Sep 15;12(9):e1461. doi: 10.1002/cti2.1461 (PMC10503407; doi:10.1002/cti2.1461)
Supplement: Supplementary file 1 — Supplementary figures 1‐5 [file CTI2-12-e1461-s002.pdf]

Autoimmune uveitis in Behçet's disease and Vogt-Koyanagi-Harada disease differ in tissue immune infiltration and T cell clonality

Supplementary information

- Supplementary figures 1–5
- Supplementary table 1 (Excel table)

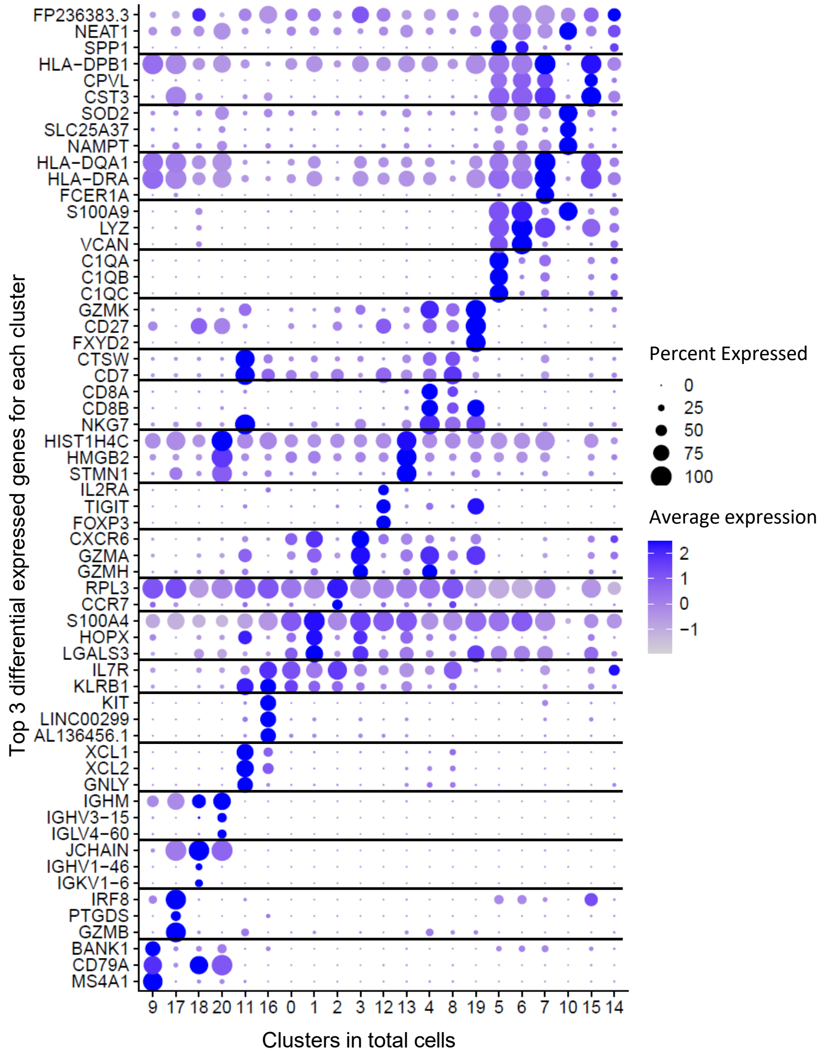

Supplementary figure 1 Dot plots of Top 3 most upregulated genes for each cell cluster of immune cells in aqueous humour of uveitis patients.

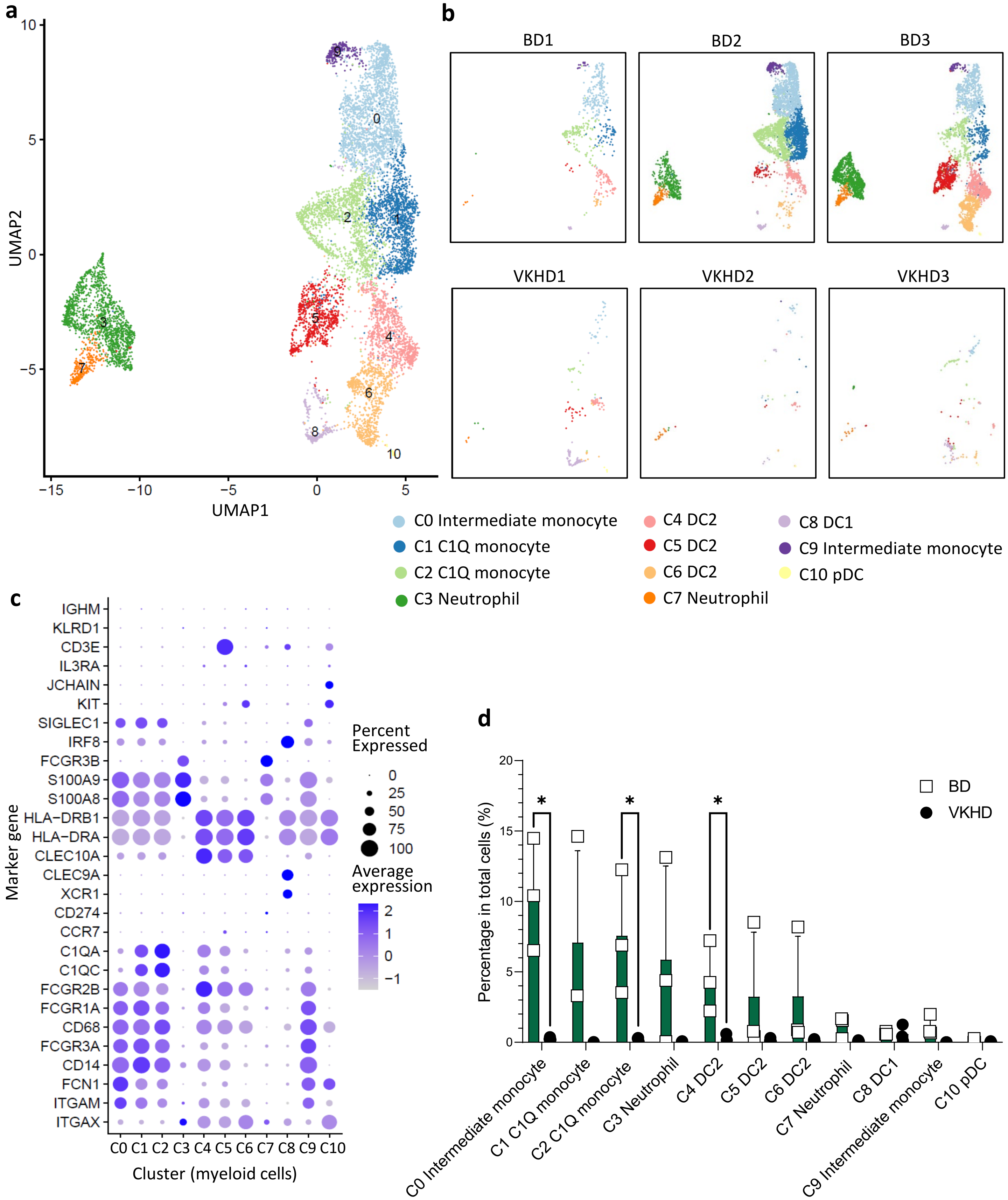

Supplementary figure 2 **(a)** UMAP plot showing unsupervised clustering of myeloid cells. **(b)** UMAP plots showing single cells and clusters of myeloid cells from each patient sample **(c)** Dot plots showing the expression of common marker genes for different myeloid cell type for cell type annotation. **(d)** Bar chart of cell types distribution for VKHD and BD, with *t*-test significance level annotation (* *P*-value <0.05)

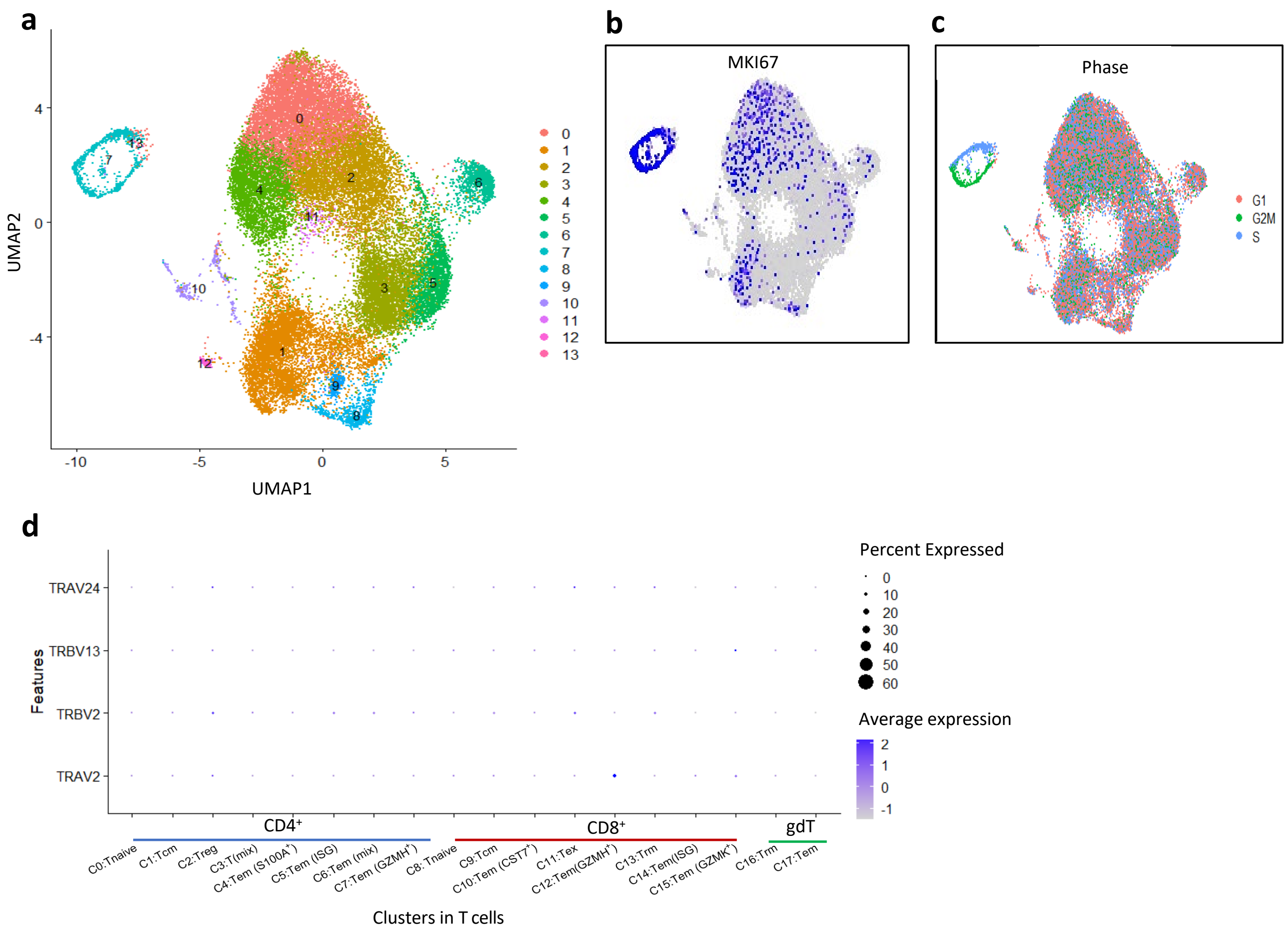

Supplementary figure 3 **(a)** UMAP plot showing original unsupervised clustering of T cells. **(b)** UMAP plot showing gene expression distribution for T cells. **(c)** UMAP plot showing cell cycle distribution in T cells. **(d)** Dot plots of four NKT and MAIT correlated TCR V genes (TRAV24, TRBV13, TRBV2 and TRAV2) expression in each T cell cluster.

a

HLA class	BD1	BD2	BD3	VKHD1	VKHD2	VKHD3
**A-1**	A*31:20	A*01:01:87	A*11:01:06	A*11:01:06	A*01:01:87	A*24:368
**A-2**				A*31:111		
**B-1**	B*15:412	B*14:02:01:19	B*40:40	B*15:12:03	B*14:02:01:19	B*40:12:01
**B-2**	B*51:13:01		B*48:03:01:02	B*35:361		B*56:67
**C-1**	C*01:30	C*08:73	C*08:03:01	C*01:51	C*08:22:01:01	C*01:51
**C-2**	C*14:02:03	C*14:06			C*15:02:33:01	
**DPA-1**	DPA1*01:03:01:34	DPA1*02:01:01:15	DPA1*02:02:08	DPA1*01:03:17	DPA1*01:03:01:34	DPA1*02:02:02:05
**DPA-2**	DPA1*02:01:01:15	DPA1*02:30	DPA1*02:12	DPA1*02:06		DPA1*02:12
**DPB-1**	DPB1*14:01:01:01	DPB1*01:01:01:03	DPB1*05:01:01:11	DPB1*05:01:01:11	DPB1*22:01:01:01	DPB1*22:01:01:01
**DPB-2**	DPB1*81:01:01:01	DPB1*38:01	DPB1*19:01:01:03	DPB1*41:01:01:01	DPB1*677:01	DPB1*665:01
**DQA-1**	DQA1*01:25	DQA1*03:02:01:02	DQA1*01:03:01:09	DQA1*03:01:01:01	DQA1*03:03:01:03	DQA1*01:03:01:09
**DQA-2**	DQA1*03:13					
**DQB-1**	DQB1*03:03:02:04	DQB1*04:04	DQB1*06:01:01:01	DQB1*06:48:01	DQB1*04:01:01:02	DQB1*06:01:01:01
**DQB-2**	DQB1*03:03:02:04					
**DRB-1**	DRB1*09:01:02:03	DRB1*04:04:01:01	DRB1*08:03:02:01	DRB1*04:05:01:01	DRB1*04:02:01	DRB1*14:05:01:01
**DRB-2**	DRB1*15:02:02:01					

b

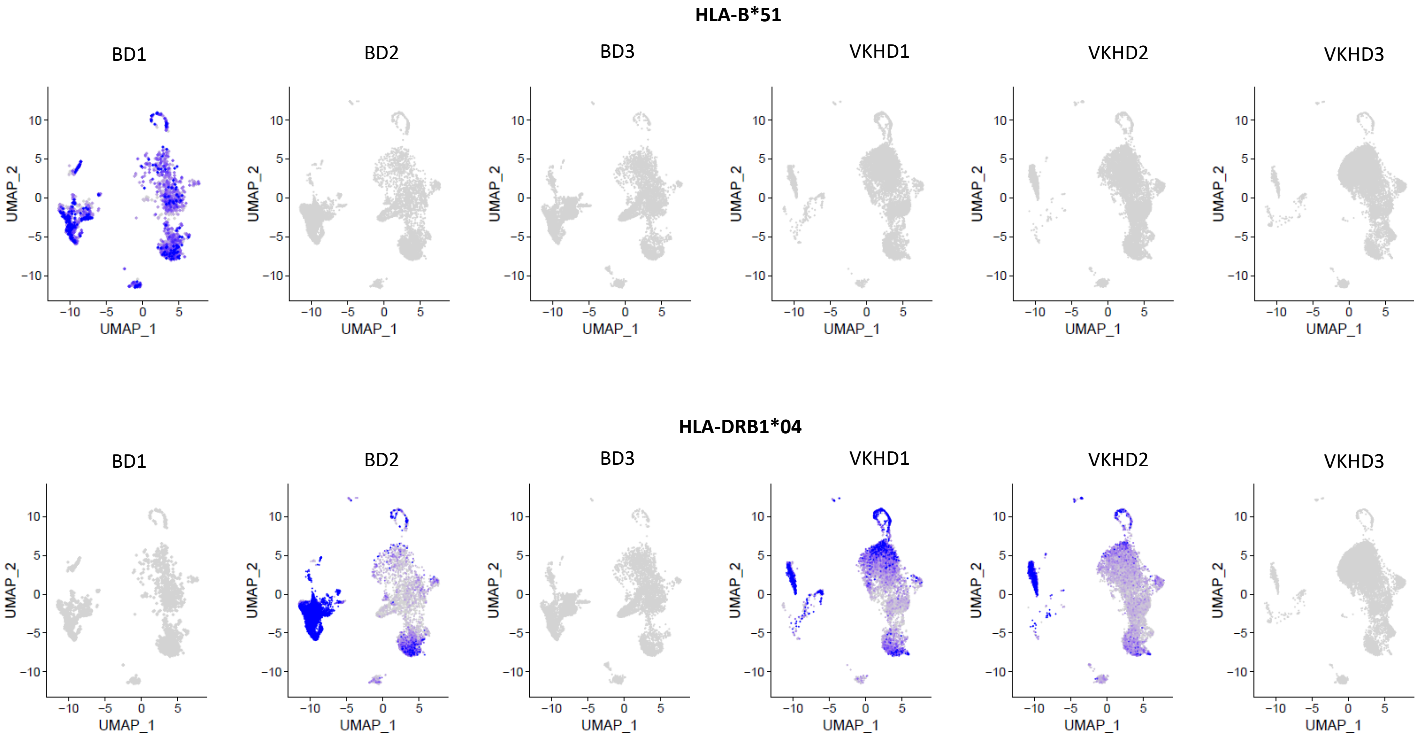

Supplementary figure 4 **(a)** Table showing each patient's HLA typing summary. **(b)** UMAP plots showing HLA-B*51 and HLA-DRB1*04 distribution. Red means high expression ,green means low expression while brown means 0 value.

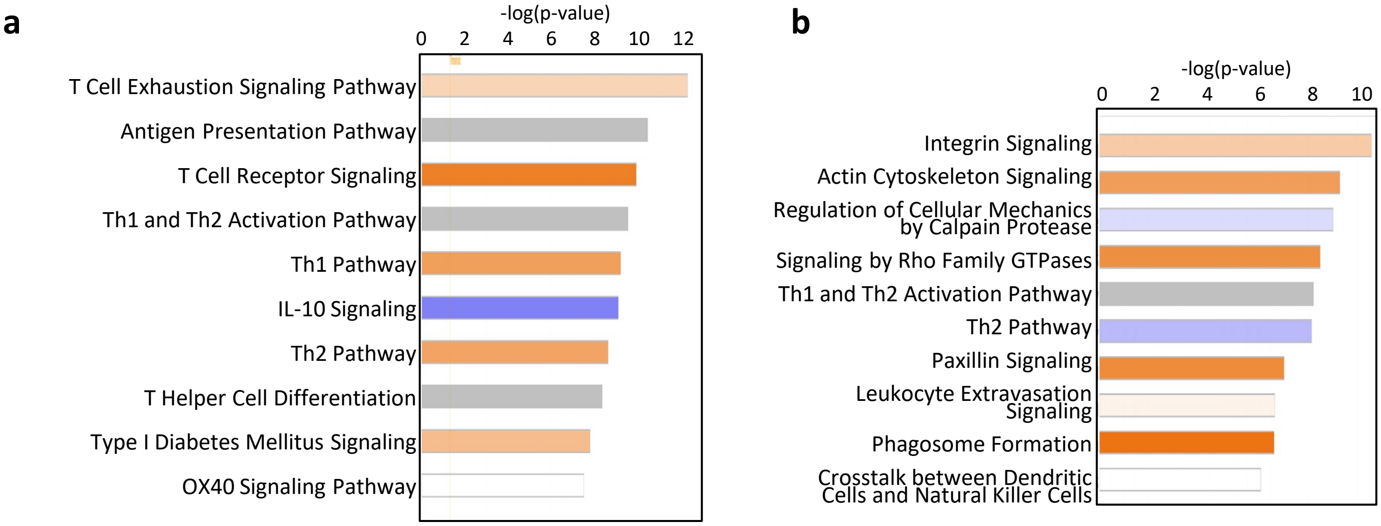

Supplementary figure **5 (a, b)** IPA pathway barplot showing significantly influencing pathways sorted by *P*-value when comparing large clonotypes (>2% T cell) with other clonotypes in $CD4^+$ **(a)** and $CD8^+$ T cells **(b).** Color reflects z-score, showing activated(orange) or inactivated (blue) or difficult to judge (white and gray).
